# Gas Detection and Classification Using Multimodal Data Based on Federated Learning

**DOI:** 10.3390/s24185904

**Published:** 2024-09-11

**Authors:** Ashutosh Sharma, Vikas Khullar, Isha Kansal, Gunjan Chhabra, Priya Arora, Renu Popli, Rajeev Kumar

**Affiliations:** 1 Business School, Henan University of Science and Technology, Luoyang 471300, China; 2 Department of Informatics, School of Computer Science, University of Petroleum and Energy Studies, Dehradun 248007, Uttarakhand, India; 3 Chitkara University Institute of Engineering and Technology, Chitkara University, Rajpura 140401, Punjab, India; vikas.khullar@chitkara.edu.in (V.K.); isha.kansal@chitkara.edu.in (I.K.); priya.sadana@chitkara.edu.in (P.A.); renu.popli@chitkara.edu.in (R.P.); 4 Department of CSE, Graphic Era Hill University, Dehradun 248007, Uttarakhand, India; chhgunjan@gmail.com

**Keywords:** dataset, gas leakage, multimodal dataset, thermal camera, image enhancement, low-cost sensors

## Abstract

The identification of gas leakages is a significant factor to be taken into consideration in various industries such as coal mines, chemical industries, etc., as well as in residential applications. In order to reduce damage to the environment as well as human lives, early detection and gas type identification are necessary. The main focus of this paper is multimodal gas data that were obtained simultaneously by using multiple sensors for gas detection and a thermal imaging camera. As the reliability and sensitivity of low-cost sensors are less, they are not suitable for gas detection over long distances. In order to overcome the drawbacks of relying just on sensors to identify gases, a thermal camera capable of detecting temperature changes is also used in the collection of the current multimodal dataset The multimodal dataset comprises 6400 samples, including smoke, perfume, a combination of both, and neutral environments. In this paper, convolutional neural networks (CNNs) are trained on thermal image data, utilizing variants such as bidirectional long–short-term memory (Bi-LSTM), dense LSTM, and a fusion of both datasets to effectively classify comma separated value (CSV) data from gas sensors. The dataset can be used as a valuable source for research scholars and system developers to improvise their artificial intelligence (AI) models used for gas leakage detection. Furthermore, in order to ensure the privacy of the client’s data, this paper explores the implementation of federated learning for privacy-protected gas leakage classification, demonstrating comparable accuracy to traditional deep learning approaches.

## 1. Introduction

Engineering design innovations are helping humanity in solving economic and societal issues. Chemical firms are using technology to solve numerous issues; however, industrial risks might harm the ecology in the area. A frequent issue in the chemical industries is gas leakage. Industrial disasters frequently occur because of explosions, leaks, waste emissions, fires, etc. The improper disposal of household waste also causes leaks of toxic gases and odors. Wood burning is another significant contributor to air pollution. Workers’ deaths have also been caused by the gases that spilled during mining operations. Even though the machines are tested for gas leaks before installation, instances of gas leakage have been documented [1]. Gas-detecting sensors are frequently used close to regions where leaks are likely to occur. However, the sensors can sense only one particular gas, and they are not able to detect leaks when a combination of gases is found. Due to the hazardous nature of chemicals, manual gas identification using chemical apparatus is never a workable solution; for instance, smoke leaks impair visibility. Because of this, in gas leak scenarios, automatic gas identification is crucial. Both human and machine lives are saved as a result.

Utilizing sensor data fusion techniques to aggregate and compress the amount of data analyzed is one such solution. Data fusion is the process of combining information and data from various sources [2]. The objective is to blend data from several sources to create a cohesive picture of the application or job at hand. The application of data fusion techniques has a number of benefits, including increased data availability and authenticity, a reduction in duplicate data exchange, and a reduction in the amount of energy used to transmit the data [3]. As a result, given the basic design of gas leak detection systems, they represent a promising solution.

Federated learning (FL) is another paradigm that is worth utilizing. Using the FL machine learning paradigm, a high-quality centralized model is trained using data that are scattered across several locations [4,5,6]. The phrase originated with Google, which, in 2016, announced a method that could use data from various sources to compute an update to the current machine learning (ML) model [4] independently. After that, a central service receives this update, collects it into a novel hybrid model, and distributes it to the different sites. Therefore, rather than “bringing the data to the code,” this paradigm supports “bringing the code to the data” [5]. As a result, issues with data ownership, privacy, and location are resolved by the FL paradigm. FL appears to be a possible method for deriving relevant information from the acquired data while retaining its privacy and localization This is as a result of the fact that gas leak monitoring systems are spread and sensors are gathering data at a number of different places.

The following are the key contributions of this paper:

1. Using a CSV dataset, the identification of gas leaks based on fundamental deep learning models is performed.
2. Using a thermal image data set, the identification of gas leakage based on fundamental deep learning models is performed.
3. Identification for a multimodal gas leakage scenario using data from both CSV and image datasets is implemented.
4. An implementation of a gas identification method utilizing FL is established to ensure the confidentiality of the consumers’ personal data.

Additionally, the structure of this document is as follows: The details and connections to the current sensor-based gas datasets are provided in Section 2. Section 3 provides a thorough explanation of the suggested multimodal gas data and how they will be collected. Section 3 also includes a link to the dataset for download. The paper is finally concluded in Section 4.

## 2. Literature Review

Gas detection and classification play crucial roles in ensuring safety and security across various industrial and environmental contexts. Several approaches are used in the literature to identify gas leakage detection. A variety of inexpensive sensors have been developed in recent years to enable Internet of Things (IoT)-enabled systems that can detect leakages of gas [7,8,9,10]. However, because low-sensitivity sensors are used, these systems have restricted possibilities. It is a difficult undertaking that calls for the employment of technology to detect gas in a mixed environment. Popular chemical methods for identifying specific gas amounts in a mixed environment are colorimetric tape and gas chromatography [11,12,13]. Khalaf suggests the least-squares-based approach for categorizing gases and calculating the gas concentration [14]. For the goal of gas detection, machine learning techniques are used in [15]. For precise gas detection applications, deep-neural-network-based approaches are also used in [16,17,18,19]. All of these papers’ approaches, however, are predicated on the input data from various gas-detecting sensors. In experiments, a variety of sensors that are sensitive to various gases are created and taken into consideration. These techniques are elaborated in Table 1, as given below:

There are certain issues related to the recognition and detection process relying upon gas sensors. In general, there are not many gases in the air, and sometimes they cannot even be detected by a typical set of gas sensors. As a result, the framework’s detection accuracy suffers and detection becomes ambiguous. Additionally, less expensive sensors are typically less accurate and may not provide precise estimates. The temperature in the immediate area rises when there is a leak; the thermal camera [20] can also detect this temperature shift. The benefit of being able to locate the gas leaking from a further distance is provided by the use of a thermal camera. The demand for an accurate training dataset is growing as a result of the increased usefulness of data analytics and artificial intelligence techniques. The dataset’s accessibility will aid in system training as well as serve as a foundation and platform for the creation of new datasets. The sensor array is the primary method used to collect the existing datasets to detect gas leakages.

The identification of gas leaks has also been carried out using thermal imaging [21,22]. Little research has used it to accomplish this goal, however. Machine learning models have been used to analyze infrared (IR) thermal pictures for detection of gas leakages, for example, in [23]. Tensor-based leakage detection (TBLD), a method for finding gas leakage in remote regions using thermal cameras, is suggested in [24]. In the classification stage of leakage, various classification techniques are examined. To precisely identify gas leaks, a residual network with 50 layers was used (ResNet50). The study [25] proposed a novel biochar adsorbent with bimetal-doping demonstrated high Hg0 removal capabilities. The adsorbent injection method using electrostatic precipitators and fabric filters shows promise in reducing mercury emissions. The Hg0 removal amount of modified biochar is 13 times that of unmodified biochar.

However, only a small number of research studies have considered the E-nose’s numerous gas sensors and multimodal fusion of thermal images. Compared to 93% and 82% accuracy of the individual modalities of gas sensor data and thermal pictures, respectively, the authors in [26] found that accuracy achieved using the multimodal fusion of gas sensor data and thermal images was found to be 96%. The study [27] also used the multimodal fusion of sensor data and thermal imaging to find gas leaks. The study’s authors contrasted intermediate fusion and multitasking techniques. The findings showed that multitask fusion is more dependable and accurate than intermediate fusion. The accuracy of the fusion model is higher than that of separate models since it uses data from both modalities.

Based on the above literature study, the following gaps are identified:

(a) Traditional gas detection systems, predominantly based on microcontrollers and IoT technologies, offer reliable means of detecting gas leakages but often face limitations in scalability and real-time monitoring capabilities [7,8,9].
(b) Techniques such as gas chromatography and electronic nose systems provide accurate analysis, but they may struggle with detecting multiple gases simultaneously or handling complex environmental conditions [11,12,14].
(c) Moreover, advancements in machine learning, particularly deep learning approaches, show promise in enhancing detection accuracy and robustness [16,17,18]. However, existing studies often focus on single sensor modalities and fail to fully leverage the potential of multimodal data integration for improved detection performance [19,26].
(d) Additionally, concerns regarding data privacy and security in centralized machine learning approaches hinder the widespread adoption of these techniques in sensitive industrial settings [20]. Therefore, there is a need for innovative methodologies that can address these gaps and provide scalable, privacy-preserving solutions for accurate and robust gas detection and classification.

The proposed methodology integrates federated learning to address the challenges of scalability and data privacy. The various contributions of the proposed work are described as follows:

1. Novel Multimodal Dataset: The authors have proposed the novel, first-of-its-kind multimodal dataset for gas detection and introduced both the numerical data collected from the gas sensors and the thermal images. The dataset consists of 6400 samples across four classes: fragrance, smoke, aerosol mixture, and smoke–aerosol mixture, and ordinary setting.
2. Multimodal Deep Learning Approach: The research incorporates gas sensors with thermal cameras in a multimodal deep learning framework that the authors developed to optimally detect and distinguish gas leaks. The first set of models includes CNNs, LSTM, and Bidirectional LSTM, which is trained and tested on multimodal datasets.
3. Federated Learning Implementation: Thus, in response to the data privacy and scalability issues, the authors use federated learning methodologies. FL allows for training models cooperatively on the data present on distributed devices without compromising data privacy, as data holders can only upload a portion of their data to the server while the model can still be improved using the wisdom of crowds.
4. Comprehensive Evaluation: The proposed multimodal and federated models are thus thoroughly assessed based on metrics like accuracy, precision, recall, loss, etc. The results show a higher accuracy of the methods being proposed in comparison with single-modality approaches and other studies using the same dataset.
5. Competitive Performance: The above presented approach is more efficient than the contemporary research, which examined the same dataset for detecting the gas leakage, since the federated learning hit rate was 0.985 and 0.992, which is higher than the accuracies presented in the prior research findings.
6. Industry 5. 0 Relevance: In the context of Industry 5, the study suggests that advances in machine learning models such as multimodal data analysis and federated learning can benefit Industry 5 applications, which demonstrates the growing demand for data-oriented control and security systems. The proposed approach ensures the provision of the much-needed ways and means for efficient, accurate, privacy-conscious, and scalable solutions for the detection and identification of gases leakage in industries.

In sum, this work introduces a new multimodal dataset, the deep-learning-based system of utilizing multiple modalities, and the federated learning deployment for gas leakage detection and its classification, which is potentially beneficial to develop the data-driven smart system for industrial safety and security domains. Top of form.

## 3. Proposed Work

**Figure 1:**
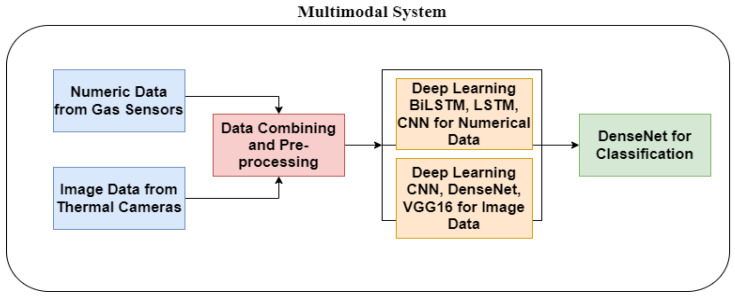
Data classification of multimodal system.

**Figure 2:**
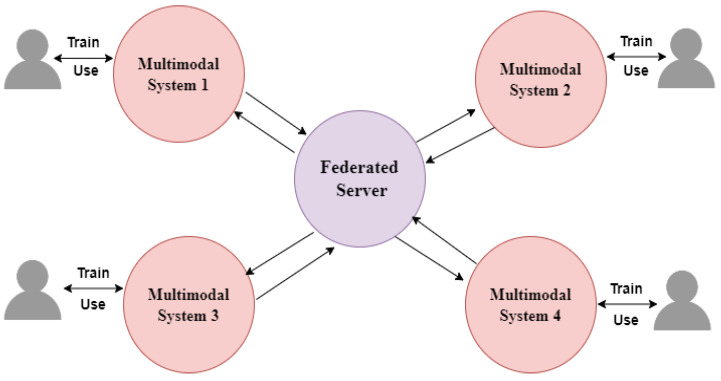
Proposed multimodal federated system.

The research outlined in this study has four primary stages for identifying gas leakage: (1) Pre-processing of data from gas sensors and thermal cameras, (2) Classification of data using a multimodal system for deep learning techniques. (3) Training and analyzing results using CNN model architectures, and (4) implementing federated learning (FL) by establishing a central server and client site for processing multimodal input. Figure 1 illustrates the data classification scheme of the multimodal system. The FL design depicted in Figure 2 showcases many architectural configurations, with the left side representing its implementation within a single facility and the right side representing its implementation across multiple facilities.

### 3.1. Dataset

For the current research, a novel multimodal dataset that was gathered utilizing thermal imaging technology and gas sensors is utilized. The following is a list of the major features of the current dataset:

- The dataset is recorded using two modalities: numerical values collected from gas sensors and images captured by the thermal camera.
- Four classifications, namely, perfume, smoke, mixes of smoke and perfume, and neutral environment, are derived from a dataset that is collected using two gas sources, which are smoke and perfume.
- This dataset is believed to be the first of its kind in gas detection and is offered for free usage.

**Figure 3:**
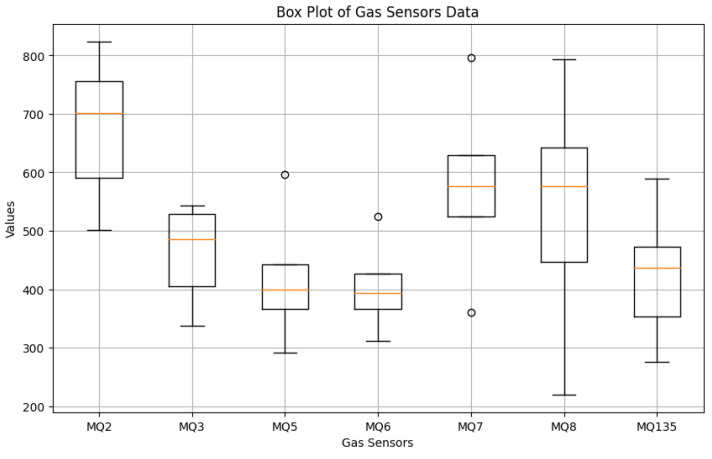
Statistical Properties of the Measurements from Gas Sensors.

The total number of samples in the multimodal gas data dataset is 6400. Four classes comprising 6400 samples are equally distributed. The dataset includes 1600 samples for the perfume class, 1600 samples for the smoke class, and 1600 samples for a combination of the two classes, as shown in Table 2 and Table 3. For the neutral environment (No gas) class, the remaining 1600 samples were gathered. The statistical analysis is also conducted to highlight the variation in the created dataset, and the results are given as a box plot in Figure 3, along with the statistical properties for the data acquired from gas sensors.

### 3.2. Multimodal Gas Detection Dataset

The dataset that was gathered utilizing numerous gas detectors and a thermal camera is the subject of the current study. In order to create a multimodal dataset, the gas sensors and thermal camera are utilized in tandem to gather information on the presence of a gas.

#### 3.2.1. Gas Sensors

**Figure 4:**
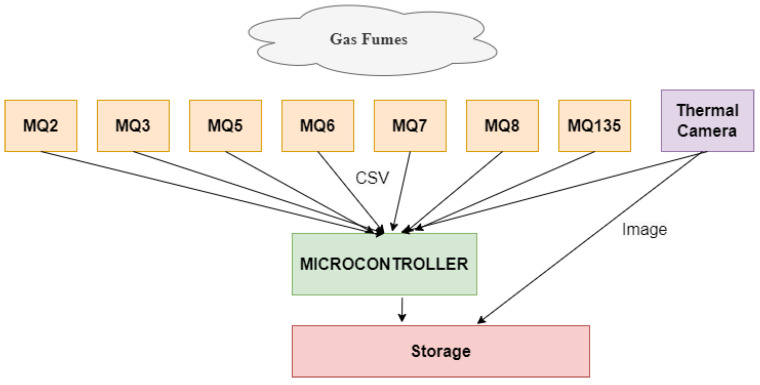
Block level connections for dataset collection setup [27].

Seven metal-oxide gas detecting sensors and a thermal camera make up the apparatus utilized for collecting the dataset. The data collecting framework is depicted in Figure 4. There are several sensors used, including a thermal camera, Sensor2, Sensor3, Sensor5, Sensor6, Sensor7, Sensor8, and Sensor135. These sensors are sensitive to a number of gases, including carbon monoxide, methane, butane, LPG, alcohol, smoke, and others, as depicted in Table 2.

#### 3.2.2. Thermal Camera

A thermal camera is a tool that uses infrared light to measure temperature fluctuations. Every pixel on a camera’s image sensor functions as an infrared temperature sensor and simultaneously measures the temperature of every point. The photos are produced using a temperature-based format and are displayed as red, green, and blue (RGB). In contrast to conventional image cameras, thermal cameras may operate in any environment, regardless of its shape or texture, and are not limited by dark environments [28]. The thermal camera that was employed in this study has a 36-degree field of view, a measurement range of 40 °C to 330 °C, a frame rate of 9 Hz, and 32,136 thermal pixels to enable easy viewing of a thermal image. It has 206,156 thermal sensors. The data for training and testing of the created fusion model are collected simultaneously using the thermal camera and gas sensors. The gathering of data and their preprocessing are covered in detail in the following section of the paper.

### 3.3. Preprocessing of Multimodal Data

The gas measurements from the seven metal-oxide (MOX) sensors are initially converted into heatmap images. Put simply, every numerical gas measurement taken every 2 s is converted into a heatmap RGB image. Each numerical value is assigned a color intensity value on the RGB scale. The measurements are plotted and assigned to a colormap pattern, resulting in the creation of an RGB image. This image is then saved with the file extension .jpg. Subsequently, the photos and IR thermal images are resized to match the dimensions of the input layers in the six distinct CNN versions. The data are subsequently partitioned into training and testing sets, with a ratio of 70% for training and 30% for testing or validation. The augmentation phase is vital for improving the training performance of CNNs. Augmenting the training data by increasing the number of photos enhances training efficacy and mitigates overfitting.

### 3.4. Data Classification for Multimodal System for DL Models

**Figure 5:**
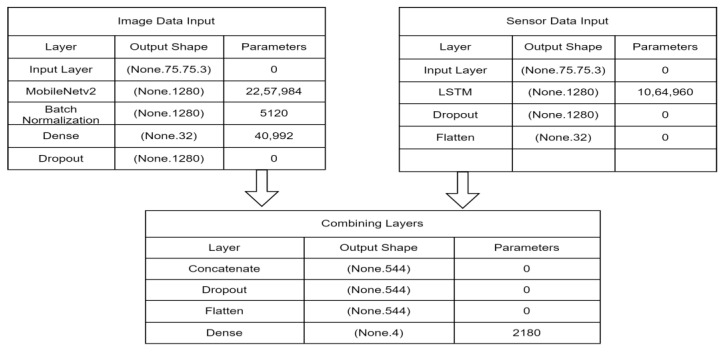
Configuration parameters of multimodal system.

During this stage, it is necessary to collect data regarding the existence of a gas in order to generate a multimodal dataset. For this purpose, the thermal camera and gas sensors are merged. We retrieve the relevant data from each data type, such as quantitative data from gas sensors and visual images from heat sensors. Subsequently, the data are prepared by performing feature extraction, data normalization, and data cleaning. We construct a composite input representation for deep learning models by amalgamating these characteristics. LSTM, BiLSTM, and CNN are among the deep learning approaches that have been trained using numerical data collected from gas sensors. Standard rectified linear unit (ReLU), and Sigmoidal activation functions were employed for mentioned classification layers. The thermal sensors provide image data that are utilized to train convolutional neural networks (CNN), DenseNet, and visual geometry group (VGG16) models. Figure 5 provides a description of the setups for both datasets. To evaluate the effectiveness of our trained model in detecting gas leaks, we assess its performance on the test dataset using measures such as accuracy, precision, recall, and loss. To assess the effectiveness of the multimodal data, it is necessary to employ both slow and fast learning rates. During a slow learning rate, each modality produces a distinct set of feature representations that capture specific information related to that modality. These features are obtained by processing the features from each modality separately using specialized neural network architectures. The neural network architecture with a quick learning rate mixes or fuses data from several modalities at an early layer, even though they are initially processed independently.

### 3.5. Multimodal Data Classification in Federated Eco-System

FL, or federated learning, is an advanced method of machine learning that is becoming increasingly popular in both academic and business settings. The following text explains and discusses the fundamental mathematical ideas and techniques that form the basis of the FL paradigm. It also explores the possibilities of the FL paradigm in addressing the problem of water leak detection.

The initial presentation provides an overview of the overall structure, followed by a detailed explanation of the FL paradigm. In most circumstances, the FL architecture consists of a centralized FL server that can communicate with a group of devices that are ready to do the required FL task. The workflow consists of six primary steps [4,5]:

- The collection of devices sends out a message signaling their availability, meaning they are ready to complete a FL task.
- The FL server distributes the ML model to a subset of these accessible devices at time t_i_.
- Each device then uses the local data to develop a new local machine learning model through a training process.
- Every device transmits the updated parameters of its machine learning model, which are derived from the previously mentioned training process.
- The FL server then combines the local models to compute the updated global ML model for time ti.
- The FL server updates the global ML model and sends it to all devices.

This process is performed every round, with the FL server choosing how often to update it.

The FL paradigm aims to acquire the W matrix-representable parameters of the global ML model inside the field of mathematics. In order to do this, a portion of the total number of D_tot_ devices is sent the model $W_{t_{i-1}}$ by the FL server. Every device $D_{t_{i}}$ goes through a training procedure to establish an updated local model $W_{t_{i}}^{j}$. Each device then transmits its update to the FL server using the formula $H_{t_{i}}^{j}$ = $W_{t_{i}}^{j}$ − $W_{t_{i-1}}$. The FL server then combines these local modifications to create the following global model [4,5]:

(1) $$W_{t_{i}}=W_{t_{i+1}}+\alpha_{t_{i+1}}H_{t_{i}}$$

where $\alpha_{t_{i}}$ is the learning rate chosen by the FL server, and $H_{t_{i}}$ is the average aggregated device-shared update, given by

(2) $$H_{t_{i}}=\frac{1}{\left|D_{\mathrm{tot}}\right|}\sum_{j\epsilon D_{t_{i}}}H_{t_{i}}^{j}$$

It is worth noting that the term $H_{t_{i}}$ can be calculated as the weighted sum of the device-shared updates rather than the average for specific implementations [5].

This paradigm can be used to facilitate spread throughout numerous regions. This is appropriate for businesses with numerous manufacturing sites spread across various regions. The FL paradigm enables businesses to learn knowledge from a gas leak in one facility and apply this knowledge to other production sites, again making use of the fact that it is unusual to have many simultaneous leaks at separate locations. Each facility would, therefore, function as a FL device in this scenario by deploying a group of servers that are utilized to carry out the local training. A centralized cloud server (like the Amazon cloud service) that serves as the FL server and aggregates local models before sending back the utilized global ML models would connect all of the various facilities. As previously noted, ML detection models like SVM and artificial neural networks can be trained at the facility level with the centralized FL server delivering the global ML model after aggregation (as they have proven to be efficient leak detection methods). It should be noted that with such an architecture, it is predicted that the data would either be heterogeneous or not independently identically distributed (non-iid) due to the facilities’ varied hardware equipment capabilities or capacities. There are several strategies that can be used to address this. One strategy is to group together similar facilities and designate one of them to deliver updates on the group’s behalf [29]. Such a strategy would address both the variety of the data and the computational capability at each of the sites. A subset of the data from each facility will be shared globally as part of the “esgrssecond approach”. In this way, the data from other sites might be viewed and observed by the local models being trained at each location. For instance, Zhao et al. [30] demonstrated that sharing just 5% of the local data worldwide might considerably raise the global model’s quality. Therefore, a similar strategy can be used for the multi-facility design to guarantee a higher-quality global model at the centralized FL server.

## 4. Experimental Results and Analysis

The following section displays the outcomes of our implementation and provides details regarding the gas detection capabilities of our models. The recommended configuration for the system is shown in Table 4. The Table 5 displays the results for recall, accuracy, precision, F1 score, and loss function for every model that has been tested. We utilized accuracy as a unified numerical examination of a system’s completion to confirm its performance, while precision and recall were utilized to validate classification. Training accuracy is also known as categorical accuracy. This metric shows how well the models are categorizing the practice data [31]. An essential part of any deep neural network is the model loss function [32]. It shows how far off the actual results are from the predictions. One way to find out how bad CNN models are at making predictions from a dataset is to look at the model loss numbers [33]. We employ the test accuracy to assess the effectiveness of the models [34]. Following training, the most effective convolutional neural network (CNN) designs were chosen based on the performance indicators. We set out to improve test accuracy while reducing the model loss function [35,36,37,38]. Over the course of ten rounds, Adam optimized all models with a learning rate of 0.0001. Data from multiple instances can be accessible at the same time due to the multimodal system. In addition, there are a number of sources for the data, such as gas sensors (which supply CSV files) and thermal imaging (which supply pictures). To train models for both types of data at the same time, multimodal deep learning models are employed. The models subsequently generate results by utilizing shared categorization. 

### 4.1. Analysis of Image and Sensor Data

Data collection and analysis from individual sensors are crucial for gas leakage detection systems to function. The accurate and timely identification of potential threats rely on these personal data. We have collected thermal camera photos and data from gas sensors into a csv file so that we can find gas leaks. For gas leakage detection systems to work, it is essential to collect and analyze data from individual sensors. For the correct and prompt detection of possible dangers, these personal data are essential. In order to detect gas leaks, we have compiled csv data from gas sensors and thermal camera images.

#### 4.1.1. Result Analysis of Image Data

To identify gas in images, we used a CNN model and six pre-trained CNN models: DenseNet201, InceptionResNet, MobileNetV2, VGG16, VGG19, and Xception. Table 5 displays the six convolutional neural network (CNN) models that we listed together with their accuracy, precision, recall, and loss. The outcomes were exceptional across all models. After looking at the accuracy values, it is clear that none of the six models can be beaten by CNN. Every single one of them obtained a precision value higher than 0.99. This result indicates that the models were most correct in predicting that there would be no gas seepage, which is the case when there is no gas leakage. When compared to competing approaches, DenseNet201, VGG19, and Xception all produce better loss values.

**Figure 6:**
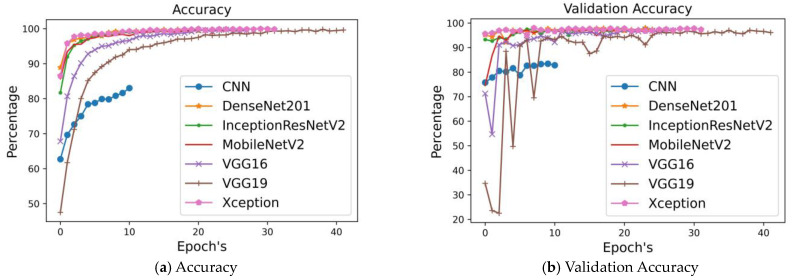

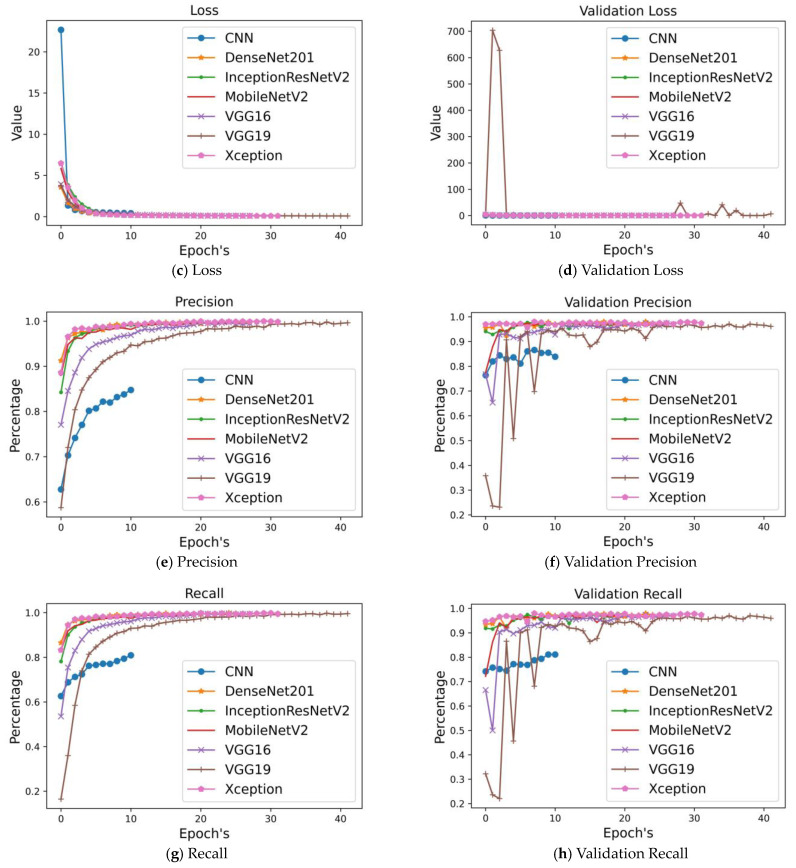
Comparing different approaches to image data validation.

Figure 6 compares the models’ performance in terms of training accuracy, validation accuracy, training precision, validation precision, training recall, validation recall, training loss, and validation loss, and it reveals that seven models perform better than the other six. A comparison of the seven designs’ training accuracy is displayed in the training accuracy graph. As can be seen, the DensNet201 and Xception models were picking up new knowledge from the training data at a respectable pace, as their upward trending lines demonstrated. At 83.000%, CNN has the lowest training accuracy (blue line). Still, each model increased its initial value from a little one. The loss functions were reduced when the models were trained for longer durations. Using test accuracy as a metric, the second figure compares and contrasts seven distinct designs. The fact that all model graphs are rising upwards in each epoch indicates that our suggested model did extraordinarily well in detecting gas leaks. The gas dataset was suitable for training our models, as shown by these results. Using the test dataset, the models accurately identified gas without being over-fit or under-fit. It is for these reasons that the models’ outstanding performance in the confusion matrix and classification report is explicable.

#### 4.1.2. Result Analysis of Sensor Data

**Figure 7:**
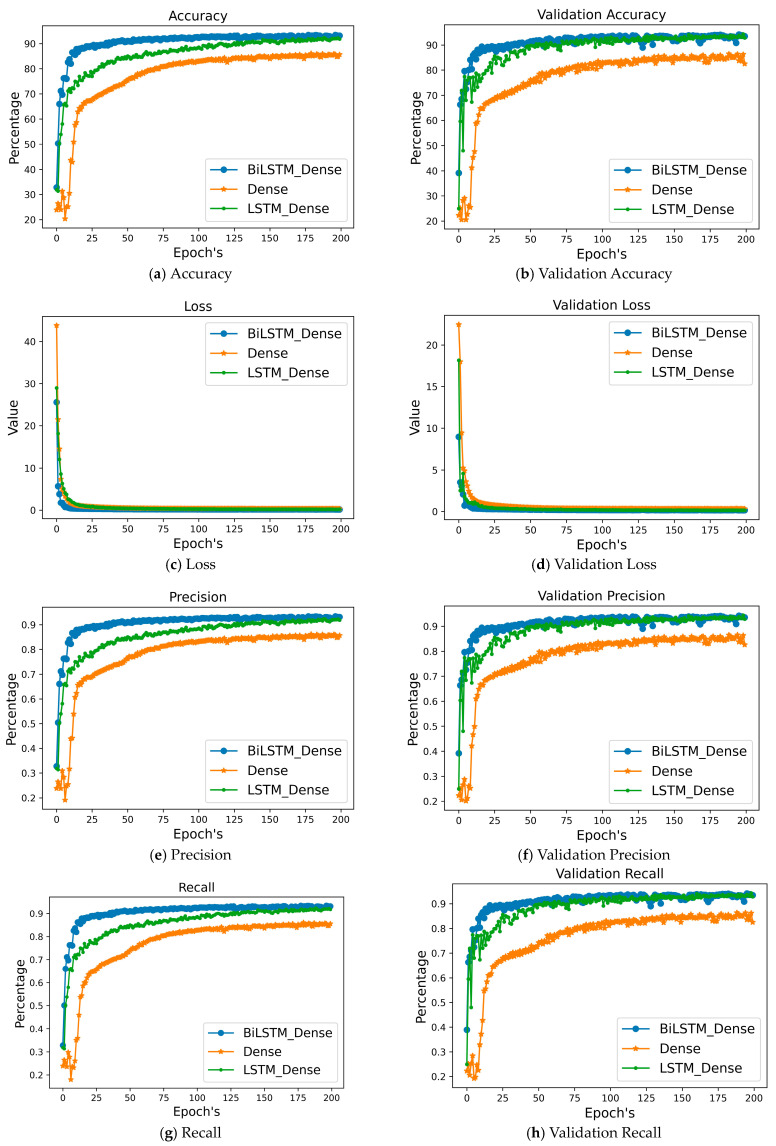
Comparing different approaches to numerical data validation.

Three deep neural networks—LSTM_DenseDenseNet201, BiLSTM_Dense, and Dense—were selected to recognize gas from CSV data. Figure 7 shows a comparison of training accuracy, test accuracy, validation loss, precision, recall, validation recall, and validation precision for these models.

Table 6 displays the results for recall, accuracy, precision, and loss for these three DL models. All of the models produced very good results. When comparing the six models’ precision values, we found that they all beat BiLSTM Dense. With a precision value of more than 93.39, each of these models was able to accurately anticipate the highest possible number of no gas leakage forecasts. With a loss value of 0.15, BiLSTM Dense is the most effective of the three methods. When it comes to detecting gas leaks, BiLSTM Dense is the best option due to its exceptional accuracy.

### 4.2. Result Analysis of Multimodal Data Using Deep Learning

**Figure 8:**
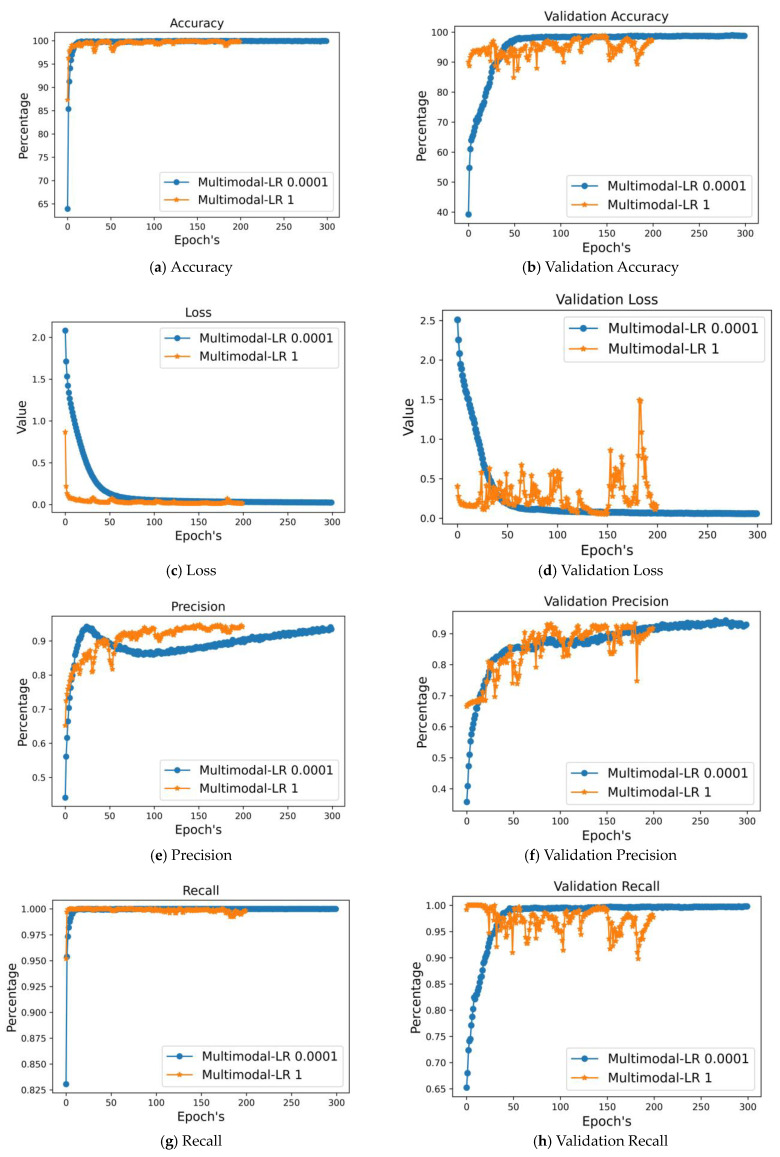
Comparing different approaches to multimodal data validation.

A precise decision was reached after combining the gas sensor results with the features extracted from thermal images. Using data from many modalities enhances the classifier’s accuracy compared to using data from a single modality alone. By utilizing multimodal representations, it is possible to train a classifier on labelled data from one modality and then apply it to data from another modality while maintaining an acceptable accuracy score. Figure 8 contrasts several metrics (training accuracy, test accuracy, recall, validation precision, validation loss, model loss function, and validation recall) to help us grasp the benefits of multimodal data. We used both slow and quick processing on multimodal data to validate our findings. According to the table, multimodal with learning rate 0.0001 (slow) performs better than multimodal with learning rate 1 (rapid) across all evaluation criteria, as shown in Table 7. Additionally, compared to single data, the accuracy and loss values for multimodal data are superior.

### 4.3. Result Analysis of Multimodal Data Using Federated Learning

Federated learning enables the collection of decentralized devices (clients) to train a model locally while retaining all training data, as opposed to transmitting data to a central server. The clients train local machine learning models utilizing their own data. After every client has finished their respective local training cycles, the central server compiles all of the model modifications that they have submitted. While protecting the privacy of individual data, this revised global model includes the expertise from all participating clients. The main benefit of federated learning for gas leak detection is that it enables the collective intelligence of all client devices to enhance the model’s accuracy without disclosing private or sensitive information.

#### 4.3.1. Implementation Details and Communication Rounds

Before selecting 10% of the clients for local training, the server engages in a maximum of six communication cycles with the clients in this scenario. Client side local training has been conducted with 100 epochs on each client with five total communication rounds between federated clients and federated server. The learning rate for the training set is 0.001.

#### 4.3.2. Performance Evaluation of Federated Learning Approach

**Figure 9:**
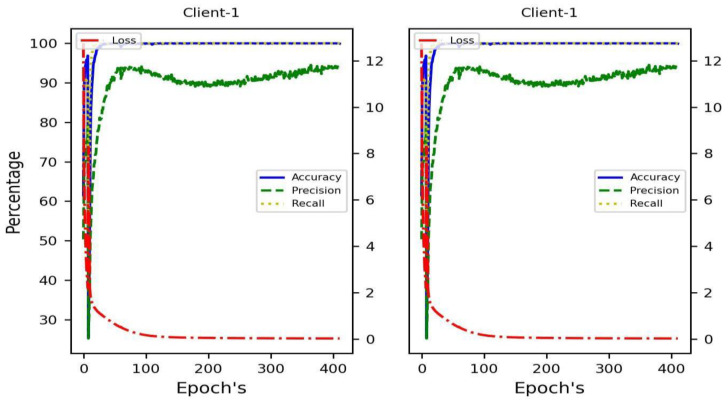

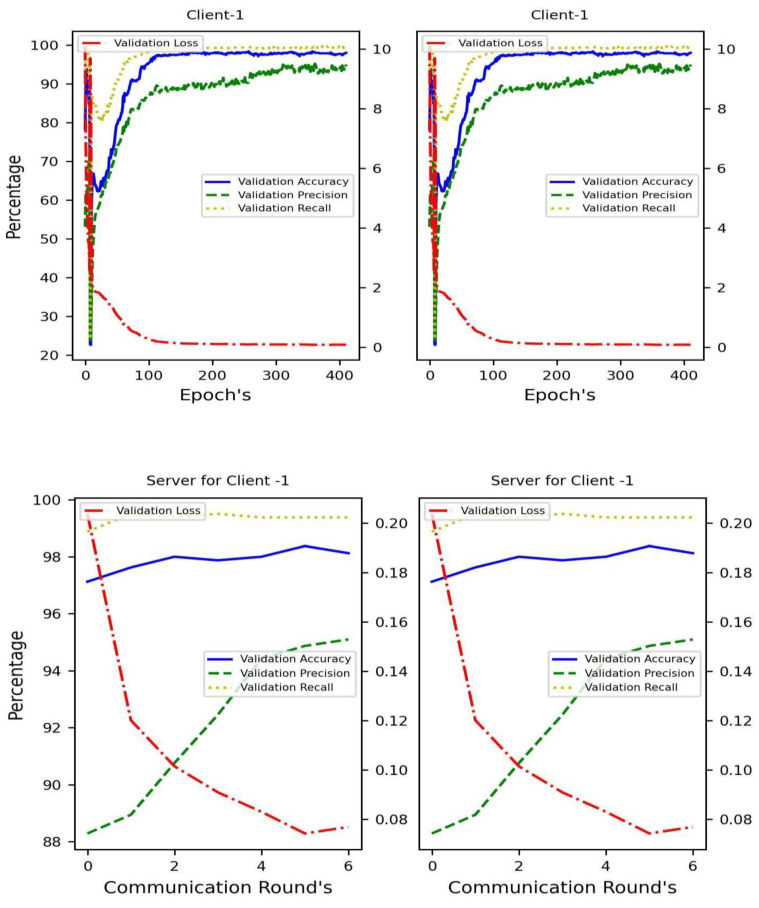
Federated multimodal aggregated results.

As illustrated in Figure 9, the aggregated validation accuracy for the multimodal data was 99.7%, and the aggregated validation loss was exceptionally low, as is evident from the results that in terms of validation accuracy and validation losses, the proposed federated multimodal system generated noticeably superior results with no additional resource requirements. The suggested federated-learning-based fake news categorization system was proven to be both much more effective and secure than the conventional frameworks and to be cost-efficient.

## 5. Discussion

A federated-learning-based multi-step approach is described for gas detection and classification using multimodal data, with the goals of improving the accuracy, privacy, and scalability of gas leakage detection systems. Data preparation and collection from gas sensors and thermal cameras is the first step in the approach. Next, the data are scaled to match the input sizes to the models, and the gas sensor values are transformed into heatmap images. The preprocessed data are subsequently classified using deep learning models such as CNNs, LSTM, and Bidirectional LSTM. At this point, the data are cleaned, normalized, and feature extracted so that they may be used as an input for the deep learning models. Recall, loss, accuracy, and precision are some of the evaluation metrics used to gauge the classification models’ efficacy. In order to train models across distributed devices without compromising data privacy, the suggested solution additionally utilizes federated learning, a distributed paradigm for machine learning. This function ensures the security of sensitive device data while utilizing collective intelligence to enhance the central model’s accuracy. Experiment parameters including learning rates, communication rounds, and local training epochs are fine-tuned to maximize the model’s performance. As the outcomes of the federated learning technique demonstrate, the suggested methodology is effective, with high validation accuracy and low validation loss. Gas leakage detection systems can be made more accurate, private, and scalable by combining federated learning with multimodal data fusion and deep learning categorization. In many commercial and domestic settings, this would improve safety protocols.

### Comparison with Existing Techniques

To illustrate the competitiveness of the proposed methodology, it is juxtaposed with other contemporary investigations that have employed the identical dataset for the purpose of gas escape detection. The results of this comparison, presented in Table 8, provide evidence that the proposed approach is superior. In contrast to similar investigations, the proposed methodology attains federated learning accuracies of 0.985 and 0.992. Significantly, its performance surpasses that of the 0.945 and 0.969 accuracies reported in [30] through the use of intermediate and multitask fusion, respectively, as well as the 0.96 accuracies derived from early fusion in [25]. This supremacy can be attributed to several factors. The proposed methodology begins with the implementation of federated learning on multimodal data. This is in opposition to previous investigations that have predominantly focused on geographical data. In the final stage of the proposed pipeline, bidirectional long–short-term memory (Bi-LSTM) is implemented for detection. It is well-known that this model outperforms the standard LSTM models that were frequently employed in prior studies, resulting in even more favorable outcomes.

## 6. Conclusions

The requirement for transporting gases or fluids (such as water or oil) from production sites to end user locations is driving a rapid increase in the design and deployment of pipelines. Many governmental and industrial stakeholders are quite concerned about finding gas leaks in these pipelines. This is because of the harms and expenses involved. Gas leaks not only result in financial and economic expenses but they also pose a safety risk, particularly in manufacturing and industrial settings.

In the industry 5.0 environment, this study presented a methodology for assessing the accuracy of intelligent multimodal data in detecting and identifying gas leaks. Since gas detector readings and infrared thermal imaging use different types of data, we compared the two to see which one was more effective for gas detection and identification. We also looked at the results of slow and rapid multimodal data. While CNN, DenseNet, and VG16 are used to train gas sensor data, LSTM, BiLSTM, and CNN are used to train temperature data for gas detection. Data that are multimodal are the result of combining the two datasets. We used slow and fast processing on multimodal data to confirm our results. According to the results, the classifier’s accuracy was enhanced when data from many modalities were used instead of just one. Given the distributed nature of gas leak monitoring systems that use sensors to gather data from different locations, FL offers a practical alternative to other methods of extracting useful insights from the collected data while also protecting its privacy and localization.

In industry revolution 5.0, data-oriented control and security systems are being developed and raised. In the case of gas identification, multimodalities of data ensure the correct identification of gas leakage. Therefore, such implementations in real-time distributed and federated ecosystems could help to enhance gas-related spillage concerns and identification.

## Figures and Tables

**Table 1:** Literature survey of various techniques.

Reference No.	Proposed Technique	Conclusion	Results
[7]	Microcontroller-based gas leakage detection	Proposed system offers a reliable method for gas leakage detection and evacuation.	Detection accuracy: 95%
[8]	IoT-based industrial plant safety system	System effectively detects gas leakages in industrial settings, enhancing safety measures.	Real-time monitoring capability
[9]	Gas leakage detection based on IoT	Utilizing IoT for gas detection provides a scalable solution with remote monitoring capabilities.	Remote monitoring via smartphone app
[10]	Gas leakage detection system using microcontroller	Microcontroller-based system offers a cost-effective solution for gas detection in various environments.	Low-cost implementation
[11]	Deep learning combined with an electronic nose	Effective in identifying gas information from soybean of different origins.	High accuracy in gas identification of over 95%
[12]	Machine learning and polarized mixed-potential gas sensors	Enhanced classification of volatile organic compounds. It demonstrates improved efficiency and reliability in sensor-based volatile organic compound (VOC) detection.	Improved classification accuracy of 92% with reduced false positive rates by 15%
[13]	Gas leak location detection using ultrasonic sensor array	Ultrasonic sensor array enables precise localization of gas leaks, facilitating rapid response measures.	Localization accuracy: 90%
[14]	Spatial-upscaling-based algorithm	The algorithm is efficient in detecting and estimating concentrations of hazardous gases. It shows high potential for use in various industrial and environmental applications.	Demonstrated detection accuracy of 90% and estimation error within 5% for hazardous gas concentrations
[15]	Multilevel interleaved group attention-based convolutional network with an electronic nose	The method provides superior gas detection performance, with enhanced accuracy and robustness. It effectively handles complex gas mixtures and varying environmental conditions.	Achieved a detection accuracy of 96% and demonstrated high robustness against sensor drift and noise.
[16]	Online drift compensation framework using active learning	The framework effectively compensates for sensor drift in real-time applications. It ensures accurate gas classification and concentration prediction over extended periods.	Maintained classification accuracy above 90% over long-term operation, with drift compensation reducing error rates by 20%.
[17]	Gas classification using deep convolutional neural networks	Convolutional neural networks enable accurate classification of different gas types, improving detection specificity.	Classification accuracy: 92%
[18]	Gas recognition using hybrid convolutional neural network (CNN) and recurrent neural network (RNN)	Hybrid CNN-RNN model offers a fast and robust approach for gas recognition, suitable for real-time applications.	real-time processing capability
[19]	Convolutional long–short-term memory neural networks for localizing gas sources	Convolutional LSTM networks facilitate autonomous gas source localization in outdoor environments, enhancing monitoring capabilities.	Source localization accuracy: 85%

**Table 2:** Sensors and corresponding sensitive gases.

Used Sensor	Gases Sensitive to Sensor
Sensor1/MQ2	Liquified petroleum gas Methane Gas Butane Gas Smoke
Sensor2/MQ3	Smoke Ethanol Alcohol
Sensor5/MQ5	Liquified petroleum gas Natural Gas
Sensor6/MQ6	Liquified petroleum gas Butane Gas
Sensor7/MQ7	Carbon Monoxide Gas
Sensor8/MQ8	Hydrogen gas
Sensor135/MQ135	Air Quality

**Table 3:** Number of sensors in each class.

Class	No of Samples
Perfume Class	1600
Smoke Class	1600
Both Perfume and Smoke Class	1600
Neutral Environment	1600
Total Samples	6400

**Table 4:** System Setup Configurations.

S.No.	Component	Configurations
1	1 Computer: Server	Processor: Core i7, NVIDIA 3070 8 GB Graphics, Memory, 32 GB RAM,
2	5 Computer’s: Clients	Processor: Core i5, NVIDIA 1650 4 GB Graphics Memory, 16 GB RAM,
3	Python	Version 3.7
4	Keras	Version 3.0
5	TensorFlow	Version 2.14
6	TensorFlow Federated	Version 1.0
7	Camera	5 MP HD
8	Gas Sensors	As mentioned in Table 2/Figure 4

**Table 5:** Comparison of different approaches to image data.

	CNN	DenseNet201	InceptionResNetV2	MobileNetV2	VGG16	VGG19	Xception
Accuracy	83.00	99.92	99.55	99.76	99.68	99.78	99.94
Validation Accuracy	83.43	97.96	97.68	97.81	97.34	97.03	97.96
Precision	84.76	99.94	99.60	99.78	99.72	99.78	99.96
Validation Precision	86.59	97.96	97.96	97.96	97.34	97.03	97.96
Recall	80.93	99.90	99.45	99.58	99.64	99.76	99.82
Validation Recall	81.09	97.96	97.96	97.81	97.34	97.03	97.96
Loss	0.40	0.07	0.11	0.10	0.08	0.07	0.07
Validation Loss	0.38	0.14	0.16	0.17	0.17	0.18	0.15

**Table 6:** Comparison of different approaches to numerical data.

	BiLSTM_Dense	Dense	LSTM_Dense
Accuracy	93.39	86.10	92.38
Validation Accuracy	94.18	86.43	94.33
Precision	93.39	86.16	92.41
Validation Precision	94.18	86.52	94.33
Recall	93.39	86.04	92.36
Validation Recall	94.18	86.37	94.33
Loss	0.15	0.29	0.17
Validation Loss	0.15	0.30	0.14

**Table 7:** Comparison of different approaches to multimodal data.

	Accuracy	Loss	Precision	Recall	Val_Accuracy	Val_Loss	Val_Precision	Val_Recall
Multimodal LR 0.0001	0.999375	0.025256	0.934398	1	0.9875	0.057837	0.929029	0.998125
Multimodal LR 1	0.9975	0.015876	0.940899	0.998333	0.973125	0.15937	0.917302	0.9775

**Table 8:** Performance comparison of existing methods with proposed method.

Autors [Ref]	Method	Accuracy	Precision	Recall	Privacy Preserve
[26]	LSTM and CNN	0.945	-	-	No
[39]	LSTM and CNN	0.969	-	-	No
[40]	Inception, DWT, and Bi-LSTM	0.985	0.985	0.985	No
[30]	Federated Deep Learning	95.61	-	-	Yes
[17]	CNN	96.67	0.96	0.96	No
[18]	PCA, XgBoost	94.17	0.92	0.94	No
	Proposed	99.7	0.94	0.99	Yes

## Data Availability

The data presented in this study are available on request from the corresponding author.
